# Assessing the completeness of suicidal poisoning surveillance systems in Northwest Morocco: A capture-recapture method

**DOI:** 10.1192/j.eurpsy.2025.2355

**Published:** 2025-08-26

**Authors:** S. Hmimou, S. Boukhorb, O. Erefai, S. Elkafssaoui, S. Irnat, N. Rhalem, M. A. Bellimam, A. Soulaymani, A. Mokhtari, R. Soulaymani-Bencheikh, H. Hami

**Affiliations:** 1 Laboratory of Biology and Health, Faculty of Science, Ibn Tofail University, Kenitra; 2 Higher Institute of Nursing Professions and Health Techniques; 3 Royal School of Military Health Service; 4 Moroccan Poison Control Center; 5 Forensic Institute of Royal Gendarmerie, Rabat, Morocco

## Abstract

**Introduction:**

Suicidal poisoning represents a significant yet frequently underreported public health concern, particularly in regions where surveillance systems fail to fully capture the scope of the issue.

**Objectives:**

This study aims to bridge this critical gap by estimating the total number of intentional poisoning cases and evaluating the completeness of the national toxicovigilance system in the Tanger-Tétouan-Al Hoceima region of northwest Morocco.

**Methods:**

This study analyzed data from suicidal poisoning cases recorded over a three-year period in the Tanger-Tétouan-Al Hoceima region. We sourced data from the Moroccan Poison Control Center (MPCC) and hospital registers in the region. The two-source capture-recapture method was employed to evaluate the completeness of the poisoning surveillance system.

**Results:**

A total of 824 suicidal poisoning cases were identified after removing duplicates, with 578 cases reported by MPCC and 286 cases from hospital records. Forty duplicates were found between the two sources. The capture-recapture method estimated a total of 4,133 cases (95% CI: 3,548-4,718), revealing that an additional 3,309 cases were not captured by the two data sources. The completeness of the surveillance was estimated at 13.98% for MPCC data and at 6.92% for hospital records.

**Conclusions:**

Despite the presence of a toxicovigilance system in Morocco, significant deficiencies remain in its completeness. There is an urgent need to enhance this system by promoting greater awareness among healthcare professionals regarding the critical importance of spontaneous reporting of intentional poisoning cases.

**Disclosure of Interest:**

None Declared

